# Influence of zinc on glycosaminoglycan neutralisation during coagulation

**DOI:** 10.1039/c8mt00159f

**Published:** 2018-08-22

**Authors:** Amélie I. S. Sobczak, Samantha J. Pitt, Alan J. Stewart

**Affiliations:** a School of Medicine , University of St Andrews , Medical and Biological Sciences Building , St Andrews , Fife , UK . Email: ajs21@st-andrews.ac.uk ; Fax: +44 (0)1334 463482 ; Tel: +44 (0)1334 463546

## Abstract

Heparan sulfate (HS), dermatan sulfate (DS) and heparin are glycosaminoglycans (GAGs) that serve as key natural and pharmacological anticoagulants. During normal clotting such agents require to be inactivated or neutralised. Several proteins have been reported to facilitate their neutralisation, which reside in platelet α-granules and are released following platelet activation. These include histidine-rich-glycoprotein (HRG), fibrinogen and high-molecular-weight kininogen (HMWK). Zinc ions (Zn^2+^) are also present in α-granules at a high concentration and participate in the propagation of coagulation by influencing the binding of neutralising proteins to GAGs. Zn^2+^ in many cases increases the affinity of these proteins to GAGs, and is thus an important regulator of GAG neutralisation and haemostasis. Binding of Zn^2+^ to HRG, HMWK and fibrinogen is mediated predominantly through coordination to histidine residues but the mechanisms by which Zn^2+^ increases the affinity of the proteins for GAGs are not yet completely clear. Here we will review current knowledge of how Zn^2+^ binds to and influences the neutralisation of GAGs and describe the importance of this process in both normal and pathogenic clotting.

## Abstract 2

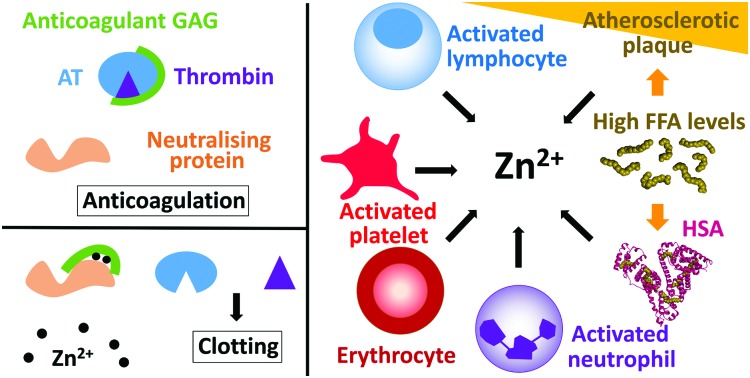
Zinc released during coagulation increases anticoagulant glycosaminoglycan-neutralisation by histidine-rich glycoprotein, high-molecular weight kininogen, and fibrinogen.

## Introduction

Glycosaminoglycans (GAGs) including heparin sulfate (HS), dermatan sulfate (DS) and heparins are key molecules involved in several biological processes, including coagulation where they play an important anticoagulant role.^1^ HS is mostly synthesised by endothelial cells, where it lines the endothelium and participates in its intrinsic anticoagulant properties.^2^ DS is mostly synthesised in the sub-endothelium and is exposed to plasma proteins during injury.^3^ Heparins are synthesised by mast cells and may also be secreted following tissue injury,^4^,^5^ however, this has been disputed by some.^6^–^8^ The main relevance of heparins are in clinical settings, where they and heparin-based drugs are important agents used in the clinical treatment of coagulatory disorders.^9^ As GAGs are physiologically found in a variety of sizes, heparin drugs are administered clinically as unfractionated heparin (UFH), which have not been cleaved or separated by size or as low molecular weight heparin (LMWH), which are generally <8000 Da.^10^ All GAGs exercise their anticoagulant activity through their binding to serpins.^1^ The main partners for HS and heparin are antithrombin (AT) and heparin cofactor II (HCII) while DS can only bind to HCII.^1^ When bound together, the GAGs can change the conformation of the reactive centre loop of the serpin to increase the inhibitory activity of the molecule.^11^ During normal coagulation, when clotting is required, anticoagulant GAGs are neutralised by several proteins, including histidine-rich-glycoproteins (HRG), high-molecular-weight kininogen (HMWK) and fibrinogen.^10^,^12^

After iron, zinc is the most abundant transition metal in the human body. Zinc is an important element in the body, playing key structural and catalytic roles as well as functioning as an extracellular and intracellular signalling molecule. Ionic zinc (Zn^2+^) is essential for physiological processes such as cell replication, tissue growth, immune functioning and coagulation.^13^–^15^ The importance of Zn^2+^ is best illustrated by the cases of zinc deficiency, which is defined as having a total plasma zinc concentration below 0.7 mg L^–1^ (normal range is 0.8–1.0 mg L^–1^).^16^–^18^ Zinc deficiency is associated with coagulatory abnormalities including a reduced ability for platelets to aggregate and longer bleeding times, which in most cases can be quickly corrected by zinc supplementation without secondary effects.^16^–^18^ In addition to the resting plasma Zn^2+^ level, during coagulation platelets release Zn^2+^ stored in their α-granules, thus initiating a signalling process.^19^–^21^ During this process Zn^2+^ acts to propagate several anticoagulation pathways as well as both pro- and anti-fibrinolytic pathways.^13^ In addition to Zn^2+^, platelet α-granules also release numerous proteins that impact on coagulation, among which are HRG, HMWK and fibrinogen.^10^,^22^ These proteins have both the ability to bind Zn^2+^ and to bind and neutralise anticoagulant GAGs.^23^–^30^ The mechanisms and impact by which Zn^2+^ influences GAG binding and neutralisation by these proteins is reviewed here.

## Zn^2+^ repartition in plasma

Despite its requirement for various physiological processes, Zn^2+^ is toxic at mid-high micromolar levels,^31^,^32^ therefore its free/labile concentration is tightly regulated. The total concentration of Zn^2+^ in the plasma is approximately 20 μM.^33^ Those ions are mostly bound to serum albumin (75% of the total Zn^2+^ concentration in the body, *i.e.* around 15 μM).^33^ The remaining Zn^2+^ (around 5–6 μM) is bound to other proteins such as α_2_-macroglobulin.^15^,^34^ This fraction is regarded as non-exchangeable as the binding is very tight.^15^,^34^ The remaining Zn^2+^ is bound to small ligands and is considered “free” or “labile” because those ligands can easily be exchanged for proteins or other ligands (more easily than when Zn^2+^ is bound to serum albumin).^15^,^34^ The free Zn^2+^ concentration in plasma is generally thought to be in the micromolar range, between 0.5 to 1 μM.^15^,^34^

**Fig. 1:**
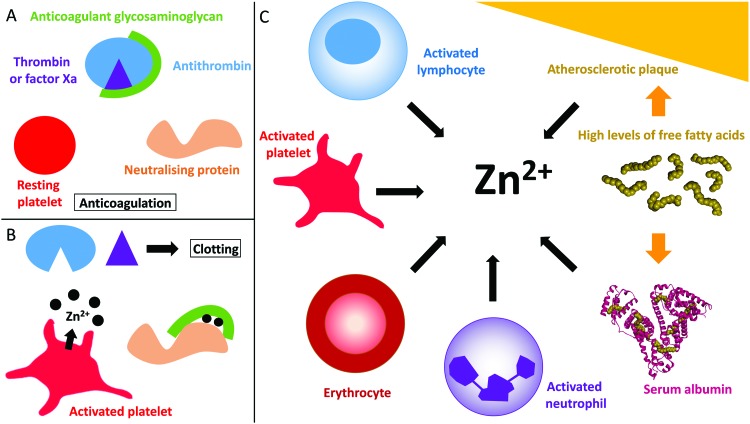
Coagulation control by glycosaminoglycans and Zn^2+^. (A) Anticoagulant glycosaminoglycans bind to antithrombin and enhance its neutralisation of thrombin (and/or factor Xa). (B) When platelets become activated, the Zn^2+^ released from the α-granules of platelets bind to the GAG neutralising proteins, increasing their affinity for GAGs and allowing them to neutralise them. Once neutralised, the GAG cannot promote the inhibition of thrombin and clotting occurs. (C) Sources of Zn^2+^ in plasma. During coagulation, Zn^2+^ is released by activated platelets. However, erythrocytes, lymphocytes and neutrophils contain Zn^2+^ which may be released under certain conditions. In some disease states, elevated levels of free fatty acids may also influence available Zn^2+^ levels through release from serum albumin. Atherosclerotic plaques contain up to six time more Zn^2+^ than healthy tissue and could potentially release Zn^2+^ when they rupture. The structure of human serum albumin (with stearate bound) was taken from PDB ; 1E7I.^109^

The proportion of free/labile Zn^2+^ in plasma is dynamic (Fig. 1). For example, during coagulation, Zn^2+^ is released from platelets.^35^,^36^ Healthy platelets accumulate around 35 g L^–1^ of Zn^2+^ that they sequester into two pools, the cytoplasm (around 60% that is used to regulate platelet function)^21^,^37^ and the α-granules (around 40%).^21^ Variations of the total amount of Zn^2+^ present in the plasma affect the quantity present in the platelets, as well as the distribution of the two pools.^37^ There is still some uncertainty as to how Zn^2+^ is incorporated into the platelets. Some Zn^2+^ may be incorporated when the Zn^2+^-bound fibrinogen-coagulation factor XIII(a_2_) complex enters the platelet through binding to fibrinogen receptors.^37^ However the main mechanism for Zn^2+^ entry into platelets is likely to be through Zn^2+^ transporters (as reviewed by Taylor and Pugh);^38^ the exact mechanism however remains to be elucidated. When platelets are activated, up to half of the α-granule Zn^2+^ pool is released.^35^,^36^ This action has been reported to increase the labile/free plasma Zn^2+^ concentration to 7–10 μM in the proximities of activated platelets.^35^,^36^ The resultant increase in Zn^2+^ concentration can then facilitate its binding to coagulatory proteins and in-turn alter their affinity for other proteins or ligands to influence the coagulation process.^19^,^20^ Platelets are not the only cells in the blood that store Zn^2+^. Indeed, neutrophils, lymphocytes and erythrocytes all contain Zn^2+^ (reported levels of total zinc are 105 μg/1 × 10^10^ cells, 116 μg/1 × 10^10^ cells and 41 μg g^–1^ haemoglobin, respectively)^39^ and may therefore release Zn^2+^ under certain circumstances in a manner similar to platelets (such as at sites of injury, although this has yet to be confirmed). In addition, the epithelium contains around 60 μg Zn^2+^/g of dry weight,^40^ and epithelial cells release some of this when damaged (the exact amount is not known).^41^

## Relevance of Zn^2+^ in coagulation control

For a long time, the role of Zn^2+^ in coagulation had been ignored and was likely often masked by the use of citrate as an anticoagulant during blood collection (with citrate forming complexes with metallic cations). However, in more recent years, the importance of Zn^2+^ in coagulation and regulation of platelet function has started to emerge.^13^,^38^,^42^ A variety of blood proteins involved in coagulatory processes have been identified as Zn^2+^-binding proteins. In many cases the ability to bind Zn^2+^ has the potential to influence their activities and impact upon haemostasis. When looking at specific interactions, Zn^2+^ has been marked as an initiator of the contact activation pathway of coagulation through enhancing the interactions of contact proteins with polyanionic surfaces and their assembly on endothelial cells and platelets.^13^ Zn^2+^ enhances platelet aggregation and activation by increasing internal platelet signalling and external binding of platelets to their ligands.^13^ It also enhances fibrin formation while also attenuating thrombin activity.^13^ Simultaneously, Zn^2+^ is also a regulator of the anticoagulant and fibrinolytic pathways. It can inhibit platelet activation by increasing HMWK and factor XII competition with thrombin to bind GPIb on platelet surface. Zn^2+^ also attenuates FXa generation by FVIIa, both increases and reduces fibrinolysis and modulates the activities of protein C, protein S and heparin-mediated anticoagulant pathways and the pro- and anti-coagulation activities of HRG.^13^

Many of the studies examining the impact of Zn^2+^ on coagulation have utilised purified protein systems where zinc-buffering or binding molecules normally found in plasma are absent. Therefore, it is not clear in some cases whether the labile Zn^2+^ concentrations used are physiologically (or pathophysiologically) attainable. With the involvement of Zn^2+^ in so many aspects of the clot process, it is difficult to tease out in which Zn^2+^ may be most involved. Dietary Zn^2+^ has been shown to exert a pronounced effect on platelet aggregation in humans and rats.^16^–^18^ Several studies have also investigated correlations between Zn^2+^ concentrations and clot formation and lysis. Generally, Zn^2+^ enhances clotting but reduces lysis – specific effects include an increase in fibrin diameter and clot porosity and reductions in clot stiffness.^36^,^43^ Yet, those studies were realised after dialysing the plasma and adding back Zn^2+^, a process which may have altered the concentration of certain (likely smaller) molecules that influence clotting.

## Zn^2+^ binding by anticoagulant GAGs

HS, DS and heparin are highly negatively-charged; thus their binding to other proteins mainly occurs through electrostatic interactions.^44^ This type of interaction will increase with the degree of sulfation of the GAGs. HS is generally less sulfated than heparin but more so than DS. Metal ions are important binding partners of GAGs and, in plasma, both Ca^2+^ and Zn^2+^ have been shown to coordinate to them.^21^ Seo, Schenauer and Leary revealed that the binding of metal ions to a heparin octasaccharide, including Ca^2+^, Mn^2+^, Co^2+^, Fe^2+^ and Ni^2+^, triggers conformational changes that have the potential to affect their interactions with their ligands.^45^ The effect of Zn^2+^ was not examined in their study but is likely to mimic the effects of these other metal ions. As both Ca^2+^ and Zn^2+^ are released from platelets during coagulation and participate in the regulation of coagulation, this is of particular interest as it is likely that this mechanism alters the anti-coagulant activities of heparin and HS following platelet activation.^21^ UFH has been shown to bind Zn^2+^*via* two different mechanisms: the first represents a high-affinity form of binding (the equilibrium constant is 976 M^–1^) whilst the second is a low-affinity form of binding that only occurs at high Zn^2+^ concentrations (the equilibrium constant is 241 M^–1^).^46^ Both binding events are entropy driven and both involve sulfated side chains on the GAGs. The exact stoichiometry of these binding events has not yet been precisely defined but it is assumed that the first mode of binding involves one zinc ion binding per disaccharide and that the second mode intervenes only after saturation is reached for the first one.^46^ GAGs interact with basic amino acids on proteins, generally lysine and arginine side-chains that are not normally affected by the presence of cations.^47^ Those cations however often bind to exposed histidines, which are positively charged, and this binding may then facilitate the binding of the protein to GAGs by reducing the electrostatic repulsion between the two of them.^47^ As Zn^2+^ is released at the beginning of the coagulation process, its effect on GAG neutralisation reduces anticoagulation and promotes clotting.

## Impact of Zn^2+^ on protein–GAG interactions

Numerous proteins in plasma have the ability to neutralise anticoagulant GAGs, as reviewed previously.^10^ Among them, three are known to bind Zn^2+^: HRG,^28^ HMWK^29^ and fibrinogen.^27^ However, the Zn^2+^ binding properties of all GAG-neutralising proteins have not been examined and there are probably more that possess this ability. This ability to bind Zn^2+^ is important as Zn^2+^ has the potential to influence GAG binding and neutralisation by those proteins (Fig. 1). HRG, HMWK and fibrinogen are stored in platelet α-granules alongside Zn^2+^ and are therefore released together during coagulation. All three proteins are synthesised in the liver and, in addition to being stored in platelets, they are present at high nanomolar to low micromolar concentrations in plasma (1.3–2.0 μM for HRG,^48^,^49^ 1–2 μM for HMWK^50^ and 12–24 μM for fibrinogen).^51^ Their specific roles in coagulation are diverse. Fibrinogen plays a prominent role as when it is cleaved, it polymerises to form fibrin clots.^52^ HRG plays a regulatory role by inhibiting fibrinolysis in addition to being incorporated in blood clots,^53^ while HMWK is a key activator of the contact activation pathway of the coagulation cascade.^54^ Thus all three proteins are in contact with endothelial GAGs and are likely to be important for GAGs neutralisation during coagulation. This section will examine the Zn^2+^ and GAG binding properties of these three proteins and how Zn^2+^ can influence GAG neutralisation.

### Histidine-rich glycoprotein (HRG)

**Fig. 2:**
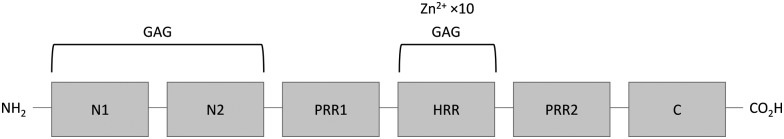
Structure of histidine-rich glycoprotein. N1 and N2 are N-terminal domain 1 and 2, they have a GAG binding activity; PRR1 and PRR2 are proline-rich regions; HRR is the histidine rich region that binds Zn^2+^ and GAG; C is the C-terminal domain.

HRG is a single chain protein composed of several structural domains that include a histidine-rich region (HRR). This region is important in both proteins, as neutrally-charged histidine residues bind Zn^2+^*via* their imidazole side chains.^28^,^48^ HRG also contains two cystatin-like domains at its N-terminus (N1 and N2) and possesses a C-terminal domain, whilst its histidine-rich region is flanked by two proline-rich regions (Fig. 2).^55^ The structure of HRG has not yet been fully resolved. A crystal structure of the N2 domain has been reported (PDB: ; 4CCV),^56^ but structural information relating to the other domains (including the Zn^2+^-binding HRR) is lacking. Nevertheless, Human HRG has been demonstrated to bind up to 10 Zn^2+^ ions with an average *K*_d_ of 6.13 μM.^48^,^57^ Current evidences suggest that there are no clearly defined preferential binding sites for Zn^2+^ on HRG.^58^ When the net charge of HRG becomes positive, either through a change in protonation of the histidine residues (through a change in pH) or through binding of those residues to metal cations, the conformation of the molecule changes, influencing its affinity for binding its ligands.^19^,^48^,^58^–^62^

HRG binds heparin with a *K*_d_ of 32.9 nM in the absence of Zn^2+^ and 5.1 nM in the presence of 1 μM Zn^2+^.^57^ Isothermal titration calorimetry studies have shown that there are two different modes of heparin binding, which are thought to occur at different binding sites.^57^ The first mode is Zn^2+^-dependent and thus most likely involves binding at the HRR.^57^,^63^ As Zn^2+^ only influences the binding of long chain heparins,^57^ this first binding site only involves long chain heparins (≥10 kDa).^57^,^64^ The second mode of binding is not dependent on chain length and is thought to occur at the N1 and N2 domains,^20^ although the exact location of this site is still unknown. The affinity of the second mode of binding is not directly affected by the presence of Zn^2+^, but Zn^2+^ binding to the HRR may induce conformation changes in HRG that would make this site more accessible to heparins. HRG forms mainly 1 : 1 complexes with heparin, but it can form 2 : 1 complexes with longer chain heparins in the presence of Zn^2+^.^64^ HRG has been shown to neutralise heparin in plasma with this ability (like binding) also dependent on the size of the heparin; with longer-chain heparins having a higher affinity for HRG.^65^ For example, even excess ratio of 500 : 1 HRG : heparin octasaccharide can neutralise less than half of the ability of heparin to accelerate the inactivation of factor Xa by AT.^65^ Zn^2+^-Dependent heparin binding by HRG only occurs when Zn^2+^ is released from activated platelets; otherwise, the metal concentration is too low and heparin preferentially binds the AT-thrombin complex.^66^ HRG can neutralise heparin-mediated thrombin inhibition by both AT and HCII, but it is much weaker in neutralising DS-mediated thrombin inhibition by HCII.^67^–^69^ HRG can also bind and neutralise HS in a Zn^2+^-dependent-manner.^70^ Thus, HRG is an important anticoagulant GAG neutraliser in plasma and this neutralisation is dependent upon the plasma Zn^2+^ concentration.

### High-molecular weight Kininogen (HMWK)

**Fig. 3:**
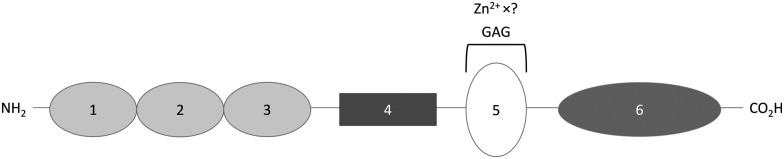
Structure of high-molecular-weight kininogen. Domains 1, 2 and 3 are cystatin-like domains, with 2 and 3 having a cysteine protease inhibitor activity; domain 4 is bradykinin and another peptide; domain 5 is the surface-binding domain containing the histidine-rich region that binds GAGs and Zn^2+^; domain 6 is the domain binding prekallikrein and activated coagulation factor XI.

HMWK is a single chain protein that consists of 6 domains, one of which, domain 5, contains a HRR (Fig. 3).^54^ Kallikrein cleaves domain 4 of HMWK, releasing bradykinin and another peptide, while the rest of the protein forms a two chain HMWK, with the heavy chain being the N-terminal section composed of domain 1, 2 and 3 and the light chain being domain 5 and 6. Both chains are then linked together by a single disulfide bond.^54^ Like HRG, HMWK has not yet been fully crystallised and so the Zn^2+^ binding domain is also not fully characterised. It is known that Zn^2+^ binds mainly to histidine residues of the HRR located between the residues Gly440 and Lys458 of domain 5. Zn^2+^ binding is known to induce a conformational change in this domain.^71^ The affinity and stoichiometry of Zn^2+^ binding by HMWK have not yet been determined despite its potential to influence the binding of HMWK to its ligands.

Like HRG, HMWK binds heparin in a Zn^2+^-dependent manner.^25^ The intact form of HMWK binds heparin with higher affinity than the cleaved form of the protein (the *K*_d_ values of intact and cleaved forms are 2.1 nM and 14.2 nM, respectively).^29^ The presence of 50 μM Zn^2+^ increases the binding affinity even further (*K*_d_ = 0.30 nM for intact HMWK).^29^ Heparin binds to the light chain portion of HMWK, at the HRR, in domain 5, which is known to mediate HMWK-binding to negatively-charged surfaces.^25^,^29^ Within this domain the binding of GAGs occurs at a combination of different sites, some of which are sensitive to Zn^2+^.^29^,^47^ In addition to histidines, this region is rich in lysine residues, which are involved in heparin binding.^29^,^47^ The heparin binding affinity of HMWK increases when the pH decreases and the histidine residues become protonated, regardless of the presence of cations.^25^ UFH and LMWH bind with similar affinity to HMWK in the absence of Zn^2+^ but the influence of the heparin chain length on the binding affinity in the presence of Zn^2+^ is not yet known.^25^ HMWK competes with AT, thrombin or the AT-thrombin complex for heparin-binding and can neutralise the anticoagulant effect of heparin in plasma.^25^ However, this binding may not be specific, as HMWK can bind all heparins regardless of whether or not they possess the saccharide sequence used to bind AT with high affinity, and thus several HMWK molecules may be required to fully neutralise one heparin molecule.^25^ Maximal neutralisation has been shown to occur in the presence of 10 μM Zn^2+^.^25^ In addition, HS proteoglycans located at the cell surface can bind HMWK in a Zn^2+^-dependent manner but the effect of this binding on the anticoagulant activity of HS has not been investigated directly.^50^ Thus HMWK appears to neutralise anticoagulant GAG in a similar manner to HRG. As HMWK is present in plasma at similar levels to HRG and binds heparin with similar affinity (*K*_d_ in low nanomolar range), these proteins may be of equal importance in anticoagulant GAG neutralisation.

### Fibrinogen

**Fig. 4:**
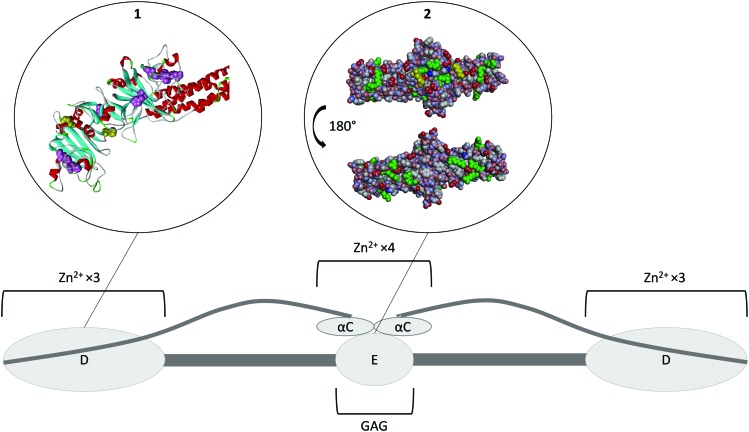
Structure of fibrinogen. The protein forms hexamer made of three different strands (Aα Bβ γ)_2_. All of the N-terminals are in the E domain which is the heparin binding domain. The three strands then coil together until they reach the D domains where the C-terminal of the β and γ strands are located. This domain is a Zn^2+^-binding domain. The α strand goes back toward the E domain where its C-terminal forms the αC domain, another Zn^2+^ binding domain. Insert 1. Crystal structure of fibrinogen D domain (one of the Zn^2+^ binding domain) and part of the coil–coil region (PDB structure ; 3GHG).^73^ The histidine residues which have the potential to be involved in Zn^2+^ binding are represented in pink with the residues His217 and His234 known to be involved represented in yellow. Most of those residues are hidden beneath the surface of the protein. Insert 2. Crystal structure of fibrinogen E domain (the heparin binding domain) and part of the coil–coil regions, (PDB structure ; 3GHG).^73^ The positive charges are represented in blue and the negative charges in red. The Lys and Arg residues which usually constitutes the main binding partners of GAGs are represented in green. The first few residues of the Bβ chain are mobile and so they are not visible in the crystal structure, with β58 the first residue that can be observed (represented in yellow). This residue is a protruding Lys that is believed to be important for GAG binding. As the GAG binding affinity of fibrinogen is enhanced when the protein is converted into fibrin by cleavage of the A and B peptides, the absence in the crystal structure of the first few residues of the Bβ chain may be the reason for exposure of the β58 residue. The αC domain is attached to the E domain; binding of Zn^2+^ ions to its His α544 and His α545 is thought to change the conformation of the protein and thus facilitate GAG binding to the E domain.

Fibrinogen is a homodimer composed of two sets of three different polypeptides chains, Aα, Bβ and γ (Fig. 4).^72^ Most of the protein has been crystallised to some extent, with the exception of the highly variable αC domain (PDB ; 3GHG).^73^ Fibrinogen binds Zn^2+^ at two different regions. The first set of binding sites is located in the D-domains (Fig. 4, insert 1) and has a stoichiometry of six (three ions per D-domain).^74^ The sites predominantly consist of histidine residues located on the γ chain, with His-γ217 and His-γ234 thought to be involved,^74^ however the effect of Zn^2+^ binding at this region on the conformation of the protein is unknown. Another Zn^2+^-binding region has been identified in the αC-domain and also involves histidine residues (His-α544 and His-α545).^75^ Binding of Zn^2+^ to this region is thought to induce a change in the conformation of the protein.^75^ Based on several studies, Zn^2+^ binding to fibrinogen has an average *K*_d_ of ∼1–18 μM, but the individual contributions of the two groups of sites are unknown.^27^,^36^,^75^

Two different heparin binding modes have been identified on fibrinogen. The first occurs at a site located on the β chain in the E domain around the Bβ1-57 region (Fig. 4, insert 2). It has been shown that a synthetic peptide corresponding to this exact region binds heparin with a *K*_d_ of 16.5 μM,^76^ whilst a dimer of the peptide, (Bβ1-66)_2_ exhibits an almost two orders of magnitude higher affinity for heparin (*K*_d_ of 210 nM), compared to the monomer.^76^ As this *K*_d_ value is close to the *K*_d_ of intact fibrinogen (228 nM), this suggests that the binding of heparin to fibrinogen occurs predominantly *via* this binding mode and that the dimeric structure of fibrinogen plays an essential role in this binding.^76^ A second heparin-binding mode occurs when Zn^2+^ binds to the αC domains of fibrinogen.^75^ Binding of Zn^2+^ is thought to induce a change in the conformation of the protein promoting heparin-binding to the nearby E domain.^75^ In presence of 12.5 μM Zn^2+^, the average *K*_d_ for heparin binding to fibrinogen is 60 nM.^75^ This represents a 4-fold increase in affinity, contrasting to a lower affinity (*K*_d_ of 539 nM) for fibrinogen without the αC domain in the presence of Zn^2+^.^75^ The link between heparin chain length and binding affinity in the presence and absence of Zn^2+^ is not yet known. The binding of fibrinogen to heparin participates in the neutralisation of its anticoagulant activity. The direct study of fibrinogen-mediated GAG neutralisation has been complicated by the fact that thrombin cleaves fibrinogen. However, fibrinogen has been shown to be more effective at neutralising DS than HRG (and platelet factor 4).^68^ This neutralisation occurs at physiological fibrinogen concentrations and is not affected by the size or the degree of sulfation of DS.^68^ The mechanism of neutralisation appears not to occur through direct competition with the thrombin–HCII complex for DS binding but by controlling the rate of formation of this complex.^68^ In addition, fibrin can also form complexes with heparin, AT and thrombin to reduce thrombin inhibition by AT in a Zn^2+^-dependent manner.^52^,^77^ Thus, fibrinogen is an important anticoagulant GAG neutraliser, with the plasma concentration of fibrinogen being linked to heparin resistance in patients.^78^ The sensitivity of fibrinogen toward plasma Zn^2+^ levels relative to HRG and HMWK is not yet known.

### Other anticoagulant GAG neutralising proteins

Zn^2+^ can also potentially affect the neutralisation of anticoagulant GAGs through, fibronectin, fibroblast growth factors (FGF), FGF-1 and FGF-7 and activated coagulation factor VII (FVIIa).^30^,^79^,^80^ Fibronectin can be found as the alternative spliced forms of cellular or plasma fibronectin. Plasma fibronectin is present in plasma at a concentration of 300–600 nM and is also stored in platelet α-granules and released during coagulation.^81^ Cellular fibronectin is synthesised by endothelial cells and can be released into plasma during wound healing.^82^ Zn^2+^ binding by fibronectin has not yet been fully characterised, however several regions have been shown to bind Zn^2+^*in vitro.* These include the collagen/gelatin binding domain (binding to which has been shown to elicit a conformational change in this region),^30^,^83^,^84^ the cell binding domain^30^ and the alternatively spliced type III connecting segment (IIICS) which is only fully present in cellular fibronectin and a small fraction of plasma fibronectin.^85^ It is still unclear whether plasma fibronectin binds Zn^2+^ physiologically or whether this only occurs with cellular fibronectin.^85^,^86^ Considering that Zn^2+^ induces a conformational change in the protein and may thus influence GAG binding, this is an important question to answer. Fibronectin possess 5–6 ionic GAG binding sites.^26^,^81^ The first binding site (often termed Hep1) is found in the N-terminal region. As Zn^2+^ binding to the neighbouring gleatin-binding region induces a conformation change in the protein, it is probable that this could impact on GAG binding in Hep1.^83^ The second GAG binding region (Hep2) has two distinct GAG binding sites and constitutes the high affinity GAG binding region.^87^ As the region is flanked by two Zn^2+^-binding regions, the cell-binding region and the IIICS, Zn^2+^ binding may also impact on GAG binding at this site. The global *K*_d_ of heparin binding to fibronectin is 0.9 μM for a 18–20 saccharide heparin (molecular weight 6000 Da).^87^ The affinity for UFH is not yet known. Fibronectin does interfere with AT binding to immobilised heparin as a function of heparin concentration. However AT is only completely displaced from heparin at fibronectin/AT ratios higher than are found physiologically.^81^ Fibronectin also binds HS and DS but its effect on their neutralisation is not known.^88^,^89^ Thus plasma Zn^2+^ levels have (at least in theory) the potential to influence GAG binding and neutralisation by fibronectin. This means that fibronectin has the potential to strongly react with the Zn^2+^ released during coagulation and to be major GAG-neutralisers during this time.

Fibroblast growth factors are present at very low concentrations in plasma (*ca.* 28–48 pM for FGF-1 and 643 pM for FGF-7),^90^,^91^ nevertheless they are normally attached to GAGs of the endothelial surface layer and depend on this binding to exert their functions (including oligomerisation, binding to their cognate receptors and transport between cells).^92^ They are therefore important binding partners for GAGs. FGF-1 and FGF-7 bind UFH with *K*_d_ values of 29 nM and 140 nM, respectively.^93^ DS also interacts with FGF-7 and FGF-1 but with lower affinity than heparin and HS. FGF-7 and to a lesser extent FGF-1 have been shown to neutralise UFH.^94^ Unlike with HRG, HMWK and fibrinogen, the affinity of these interactions are reduced by the presence of metal ions (Na^+^, K^+^, Ca^2+^, Cu^2+^ and Zn^2+^).^93^ However, it is unknown whether metal ion binding influences the neutralisation of the GAG. It is possible that binding of metal ions by these FGFs is a mechanism to facilitate their release from the endothelium when the plasma concentrations of those ions are elevated. Nevertheless, FGF-1 and FGF-7 are unlikely to be important GAG neutralisers *in vivo* due to their low plasma levels.

FVIIa is a coagulatory protein involved in the contact activation pathway.^79^,^80^ It binds UFH with a *K*_d_ value of 3.38 μM in a Ca^2+^-dependent manner.^95^ FVIIa possesses two Zn^2+^ binding sites but it is not yet known if binding of Zn^2+^ plays any role in heparin binding.^96^ FVIIa can neutralise both UFH and LMWH, but its effects on HS and DS have not yet been studied. However, like FGF-1 and FGF-7, FVIIa is only present at a low concentrations in plasma (*ca.* 16 nM)^97^ and therefore it is not likely to be as relevant in anticoagulant GAG neutralisation as the proteins listed above. Nevertheless, the interaction of Zn^2+^ with HRG, HMWK and fibrinogen shows that Zn^2+^ is an important regulator of GAG binding and neutralisation by plasma protein and it is therefore important to investigate whether Zn^2+^ has the same effect on other GAG neutralising proteins.

## Clinical significance of Zn^2+^-induced GAG neutralisation

In addition to being released during injury by epithelial cells and platelets, plasma Zn^2+^ levels are also increased in certain disease states (Fig. 1). Indeed, atherosclerotic plaques are also known to contain up to six-times more Zn^2+^ than healthy tissue.^98^ However, only total Zn^2+^ concentration has been measured and so the concentration of labile Zn^2+^ is not clear. The increase in Zn^2+^ concentration in atherosclerotic plaques correlates with an increase in Ca^2+^ concentration.^98^ In addition, both metal ions are present at high levels in areas of plaque mineralisation.^99^ This may signify that the accumulation in both metals occurs through a common mechanism that has not yet been identified. Accumulated Zn^2+^ could be released into the blood during plaque rupture, thus participating in the pro-thrombotic nature of these events.

The concentration of available Zn^2+^ is also directly influenced by the plasma free fatty acid (FFA) levels. Serum albumin is the main plasma carrier for both Zn^2+^ and FFAs.^57^,^100^ When a FFA molecule binds at a high affinity binding site (called the FA2 site) adjacent to the main Zn^2+^ binding site, an allosteric interaction leading to perturbation of the Zn^2+^ binding site occurs. In healthy individuals around 75% of total plasma Zn^2+^ (around 15 μM) is bound to serum albumin and so this mechanism may result in the release of up to 15 μM Zn^2+^ in plasma in individuals with elevated FFA levels (when those levels are elevated enough to completely prevent Zn^2+^ binding by serum albumin). In certain conditions, the concentration of FFA can increase by up to six times: in diabetes FFA concentrations have been reported to be 0.62–0.82 mM in men and 0.82–0.98 mM in women (compared to controls of 0.59–0.68 mM for men and 0.74–0.83 mM for women).^101^ In non-alcoholic fatty liver disease, the corresponding concentrations are 0.12–3.4 mM (compared to controls of 0.11–0.9 mM).^102^ In obesity, FFA concentrations are 0.56–1.15 mM (compared to controls of 0.28–0.89 mM).^103^ Elevated FFA levels are also associated with some cancers; in malignant lymphoma FFA concentrations were found to be 0.55–1.8 mM (no controls);^104^ All of these conditions are associated with a higher incidence of developing thrombotic complications.^105^,^106^ In addition, under hyperglycemic conditions, serum albumin can undergo non-enzymatic glycation reactions which can disrupt the protein conformation and also directly affect its main Zn^2+^-binding site.^107^ This is another mechanism by which diabetic state can affect Zn^2+^ transport and speciation.

Another condition that may be associated with altered plasma Zn^2+^ homeostasis is analbuminemia (albumin deficiency), which is defined as having a plasma albumin level of <1 g L^–1^. In total, 78 cases have been reported in the analbuminemia register (; www.albumin.org) and the prevalence of the disease is estimated to be less than 1 in 1 million.^108^ Individuals with analbuminemia have been reported to have elevated levels of other plasma proteins, including coagulation factors, as a compensatory mechanism.^108^ It is thought that those proteins take up most of the functions of albumin and therefore it is unclear if Zn^2+^ transport in plasma is affected or not. However, it has been shown that Ca^2+^ and Fe^2+^ have an altered protein-binding profile in these individuals.^108^ The transport of FFA is taken up by apolipoprotein B-100 and so FFA levels are close to normal (but dyslipidaemia is present).^108^ Follow-up studies of patients with analbuminemia have been limited, which limits the report of complications. Nevertheless, such follow-up studies have included reports of atherosclerosis and hypercoagulability in these individuals,^108^ but it is not clear if this may be due to alterations in plasma Zn^2+^ level/speciation or changes in plasma coagulatory protein levels.

The impact of Zn^2+^ on anticoagulant GAG-neutralisation is useful to consider, specifically as this process impacts on thrombin activation, an event that directly affects both platelet aggregation (through binding to cell surface receptors) and fibrin clot formation (through cleavage of fibrinogen). Indeed, by enhancing the neutralisation of the GAG present in the endothelium surface layer, Zn^2+^ induces a change in the natural anticoagulant properties of the endothelium.^10^ When coagulation is needed, this participates in the promotion of clotting. However, if Zn^2+^ speciation is chronically altered, such as is likely in certain diseases states (atherosclerosis, diabetes, obesity and cancer), then this could affect anticoagulant processes in the endothelium resulting in a pro- or hyper-thrombotic state through enhanced GAG neutralistaion.^10^ This phenomenon could also directly affect the efficiency of heparin-based treatments, which are widely used during surgeries and to manage thrombotic complications.^10^ In order to confirm this, it would be interesting to measure plasma Zn^2+^ levels in patients undergoing heparin treatment and to compare it to their response to this treatment. If such a relationship is confirmed then an option may be to control Zn^2+^ levels in those patients rather than to switch to another anticoagulant treatment whose efficiency could also be potentially affected by Zn^2+^.

## Conclusion

Zn^2+^ plays a major role in the regulation of coagulation that is only starting to be understood. Because of this role, Zn^2+^ homeostasis in platelets and its speciation in plasma are especially relevant to the understanding and treatment of blood diseases. In particular, they can affect GAG binding and neutralisation by the platelet-stored proteins HRG, HMWK and fibrinogen. This mechanism is relevant to healthy coagulation processes through the natural anticoagulation properties of the endothelium, but also in anti-thrombotic treatments. Indeed, it may partly explain the observed variability in dose response to heparins and heparin-based drugs. This implies that free plasma Zn^2+^ levels need to be monitored in individuals with coagulatory disorders or at-risk of thrombotic events. The control of free plasma Zn^2+^ levels may also be a treatment lead for individuals suffering from high levels of plasma FFA who are at high risk of thrombotic disorders.

## Abbreviations

AT: Antithrombin
DS: Dermatan sulfate
FGF: Fibroblast growth factor
FVIIa: Activated coagulation factor VII
GAG: Glycosaminoglycan
HCII: Heparin cofactor II
HMWK: High-molecular weight kininogen
HRG: Histidine-rich glycoprotein
HRR: Histidine-rich region
HS: Heparan sulfate
IIICS: Type III connecting segment of fibronectin
LMWH: Low molecular weight heparin
UFH: Unfractionated heparin

## Conflicts of interest

There are no conflicts to declare.
